# Supplementary material for: The prevalence of depressive disorder and its association in Thai cervical cancer patients
Source: PLoS One. 2021 Jun 21;16(6):e0252779. doi: 10.1371/journal.pone.0252779 (PMC8216533; doi:10.1371/journal.pone.0252779)
Supplement: S1 Table — (PDF) [file pone.0252779.s001.pdf]

S1 Table. The original language of the developed questionnaire was used in study.

**CASE REPORT NUMBER** ◯ ◯ ◯			
Data Record Date วันที่เก็บข้อมูล (วันที่/เดือน/ปี) ◯ ◯ / ◯ ◯ / ◯ ◯ ◯ ◯			
**แบบประเมินข้อมูลพื้นฐานทั่วไป**			
1	Birth Date เกิดเมื่อ (วันที่/เดือน/ปี) ◯ ◯ / ◯ ◯ / ◯ ◯ ◯ ◯		
2	Educational level ระดับการศึกษา<br>◯ (1) no ไม่ได้เรียนหนังสือ ◯ (2) primary school ชั้นประถมปีที่ 1-6 (1-6 ปี)<br>◯ (3) secondary school มัธยมปีที่ 1-3 หรือเทียบเท่า (7-9 ปี) ชั้นมัธยมปีที่ 4-6 หรือเทียบเท่า (10-12 ปี)<br>◯ (4) higher education ระดับมหาวิทยาลัย หรือเทียบเท่าขึ้นไป (>13 ปี) ◯ (5) others อื่น ๆ …….		
3	Marital status สถานภาพสมรส<br>◯ (1) single โสด ◯ (2) married แต่งงานหรืออยู่ด้วยกัน<br>◯ (3) separated or divorce หย่าร้างหรือแยกกันอยู่ ◯ (4) widowed คู่สมรสเสียชีวิต		
4	Number of children จำนวนบุตรทั้งหมด……………………คน		
5	Perception of external support มีผู้ดูแลหรือไม่ เช่น ครอบครัว, เพื่อน, เจ้าหน้าที่ที่เกี่ยวข้อง<br>◯ (1) no ไม่มี ◯ (2) have มี		
6	Have family history of psychiatric disorder ญาติสายตรงในครอบครัว ป่วยเป็นโรคเกี่ยวความจำหรือโรคสมองเสื่อมหรือไม่ **ประวัติจิตเวชในครอบครัว ◯ (1) have มี ◯ (2) no ไม่มี		
7	Personal income per month เศรษฐานะทางการเงิน รายได้ ต่อ เดือน<br>◯ (1) 5000 baht and less น้อยกว่าหรือเท่ากับ 5,000 บาท<br>◯ (2) 5,001 - 10,000 baht 5,001 - 10,000 บาท<br>◯ (3) 10,001 - 15,000 baht 10,001 - 15,000 บาท ◯ (4) more than 15000 baht 15,000 บาทขึ้นไป		
8	Financial problem ปัญหาเรื่องค่าใช้จ่ายเกี่ยวกับการเจ็บป่วย<br>◯ (1) severe มีมาก ◯ (2) moderate มีปานกลาง ◯ (3) mild มีเล็กน้อย ◯ (4) no ไม่มีเลย		
9	Menopause ท่านอยู่ในระยะหมดประจำเดือนหรือไม่ ◯ (1) yes ใช่ ◯ (2) no ไม่ใช่		
10	Menopausal symptoms ท่านมีอาการจากภาวการณ์หมดประจำเดือนหรือไม่ ◯ (1) yes ใช่ ◯ (2) no ไม่ใช่		
แบบบันทึกการตรวจวินิจฉัยโรคซึมเศร้าตามเกณฑ์ **Clinical diagnosis evaluating by DSM** 5<br>Psychiatrist ผู้ประเมิน..			
1	According to your evaluation, did patient have depression? จากการประเมิน ผู้ป่วยมีภาวะซึมเศร้าหรือไม่	◯ no ไม่ใช่ (1)	◯ yes ใช่ (2)
2	Diagnosis from DSM 5 ระบุวินิจฉัยตามเกณฑ์ DSM 5		
	◯ 1.adjustment(1)		
	◯ 2.MDD(2)		
	◯ 3.PDD(3) specifier		
	◯ 4.other(4) ระบุ.........................……………		
แบบประเมินเกี่ยวกับโรคและการรักษา **Disease and treatment record**			
9	First diagnosis (date/month/year) วันเวลาที่ได้รับการวินิจฉัย (วันที่/เดือน/ปี)<br>◯ ◯ / ◯ ◯ / ◯ ◯ ◯ ◯		
10	Type of cancer ชนิดของมะเร็ง<br>◯ (1) squamous cell carcinoma ◯ (2) non-squamous cell carcinoma		
11	Stage of cancer ระยะของการเป็นมะเร็งปากมดลูก<br>◯ (1) stage 1 ระยะที่ 1 ◯ (2) stage 2 ระยะที่ 2 ◯ (3) stage 3 ระยะที่ 3 ◯ (4) stage 4 ระยะที่ 4		

12	Recurrent of cancer มีการกลับเป็นซ้ำหรือไม่ ◯ (1) yes มี ◯ (2) no ไม่มี
13	Metastasis of cancer มีการกระจายของมะเร็งหรือไม่ ◯ (1) yes มี ◯ (2) no ไม่มี
14	Previous cancer treatment วิธีการรักษาที่เคยได้รับทั้งหมด<br>(1) surgery ผ่าตัด ◯ (1) no ไม่เคย ◯ (2) yes เคย<br>(2) radiotherapy ฉายรังสี ◯ (1) no ไม่เคย ◯ (2) yes เคย<br>(3) chemotherapy เคมีบำบัด ◯ (1) no ไม่เคย ◯ (2) yes เคย<br>(4) hormonal therapy การรักษาด้วยฮอร์โมน ◯ (1) no ไม่เคย ◯ (2) yes เคย<br>(5) other อื่น ๆ ระบุ .... ◯ (1) no ไม่เคย ◯ (2) yes เคย
15	Stage of treatment ระยะที่ทำการรักษา<br>◯ (1) first line อยู่ระหว่างรับการรักษาด้วย ◯ (2) second line/ third line อยู่ระหว่างรับการรักษาด้วย<br>◯ (3) annual check up ตรวจติดตามต่อเนื่อง
16	Current chemotherapy เคมีบำบัดที่ได้รับ ◯ (1) yes มี ◯ (2) no ไม่มี
**แบบประเมินโรคทางอายุรกรรมที่พบร่วมในผู้ป่วย (Charlson co-morbidity index)**	
**แบบประเมินความอ่อนเพลีย (11-item Chalder Fatigue Scale)**	
**แบบประเมินคุณภาพชีวิตของผู้ป่วยโรคมะเร็ง (EORTC QLQ-C30)**	
**แบบสอบถามคุณภาพชีวิตเฉพาะในผู้ป่วยมะเร็งนรีเวช (EORTC-QLQ-CX24)**	
**แบบประเมินอาการปวด (Numeric Pain Rating Scale for Pain)**	
**แบบประเมินความรุนแรงของภาวะซึมเศร้า (PHQ-9)**	
**แบบประเมินความรุนแรงของการฆ่าตัวตาย (8Q)**	
