# Supplementary material for: The prevalence of depressive disorder and its association in Thai cervical cancer patients
Source: PLoS One. 2021 Jun 21;16(6):e0252779. doi: 10.1371/journal.pone.0252779 (PMC8216533; doi:10.1371/journal.pone.0252779)
Supplement: S2 Table — (PDF) [file pone.0252779.s002.pdf]

S2 Table. The English version of the developed questionnaire was used in study.

**CASE REPORT NUMBER** ◯ ◯ ◯	
Data Record Date ◯ ◯ / ◯ ◯ / ◯ ◯ ◯ ◯	
**demographic data**	
1	Birth Date ◯ ◯ / ◯ ◯ / ◯ ◯ ◯ ◯
2	Educational level<br>◯ (1) no ◯ (2) primary school ◯ (3) secondary school<br>◯ (4) higher education ◯ (5) others ………………………………………………
3	Marital status<br>◯ (1) single ◯ (2) married ◯ (3) separated or divorce ◯ (4) widowed
4	Number of children ....
5	Perception of external support ◯ (1) no ◯ (2) have
6	Have family history of psychiatric disorder ◯ (1) have ◯ (2)no
7	Personal income per month<br>◯ (1) 5000 baht and less ◯ (2) 5,001 - 10,000 baht<br>◯ (3) 10,001 - 15,000 baht ◯ (4) more than 15000 baht
8	Financial problem ◯ (1) severe ◯ (2) moderate ◯ (3) mild ◯ (4) no
9	Menopause ◯ (1) yes ◯ (2) no
10	Menopausal symptoms ◯ (1) yes ◯ (2) no

**Clinical diagnosis evaluating by DSM** 5<br>Psychiatrist...			
1	According to your evaluation, did patient have depression?	◯ no	◯ yes
2	Diagnosis from DSM 5		
	◯ 1.Adjustment(1)		
	◯ 2.MDD(2)		
	◯ 3.PDD(3) specifier		
	◯ 4.other(4) ....……………		

**Disease and treatment record**	
9	First diagnosis (date/month/year) ◯ ◯ / ◯ ◯ / ◯ ◯ ◯ ◯
10	Type of cancer ◯ (1) squamous cell carcinoma ◯ (2) non-squamous cell carcinoma
11	Stage of cancer ◯ (1) stage 1 ◯ (2) stage 2 ◯ (3) stage 3 ◯ (4) stage 4
12	Recurrent of cancer ◯ (1) yes ◯ (2) no
13	Metastasis of cancer ◯ (1) yes ◯ (2) no
14	Previous cancer treatment<br>(1) surgery ◯ (1) no ◯ (2) yes<br>(2) radiotherapy ◯ (1) no ◯ (2) yes<br>(3) chemotherapy ◯ (1) no ◯ (2) yes<br>(4) hormonal therapy ◯ (1) no ◯ (2) yes<br>(5) other………………… ◯ (1) no ◯ (2) yes
15	Stage of treatment<br>◯ (1) first line ◯ (2) second line/ third line ◯ (3) annual check up
16	Current chemotherapy ◯ (1) yes ◯ (2) no
**Charlson co-morbidity index**	
**11-item Chalder Fatigue Scale**	
**EORTC QLQ-C30**	
**EORTC-QLQ-CX24**	

**Numeric Pain Rating Scale for Pain**
**Personal Health Questionnaire Depression Scale (PHQ-9)**
**Suicidal risk assessment (8Q)**
