# Supplementary material for: The prevalence of depressive disorder and its association in Thai cervical cancer patients
Source: PLoS One. 2021 Jun 21;16(6):e0252779. doi: 10.1371/journal.pone.0252779 (PMC8216533; doi:10.1371/journal.pone.0252779)

PLOS ONE

The prevalence of depression and its association in Thai cervical cancer patients

--Manuscript Draft--

**Manuscript Number:**	PONE-D-20-32916
**Article Type:**	Research Article
**Full Title:**	The prevalence of depression and its association in Thai cervical cancer patients
**Short Title:**	Depression in cervical cancer
**Corresponding Author:**	Nuntaporn Karawekpanyawong, M.D.<br>Chiang Mai University<br>A.Muang, Chiang Mai THAILAND
**Keywords:**	cervix; malignancy; depress; depressive disorder; risk; predictor; quality of life
**Abstract:**	Purpose: This study examined the prevalence, risk factors, and quality of life associated with depression in cervical cancer patients.<br>Patients and methods: This cross-sectional, observational study was carried out in the gynecologic oncology clinic of a university hospital in Northern Thailand from October 2018 to August 2019. This study included two-hundred patients with all stages of cervical cancer. We assessed all participants with the nine-item Patient Health Questionnaire (PHQ-9; Thai version), The Diagnostic and Statistical Manual of Mental Disorders Fifth Edition (DSM-5) classification, a suicidal screening form (the 8Q), the Charlson Comorbidity Index (CCI), the eleven-item Chalder Fatigue Scale (CFQ 11), the visual analog scale (VAS) for pain, the European Organization for the Research and Treatment of Cancer Quality of Life Questionnaire Core 30 (EORTC QLQ-C30) and Cervical Cancer Module (EORTC QLQ-Cx24).<br>Results: Twenty-seven (13.5%) patients were diagnosed with depression by psychiatrists according to the DSM-5. The cervical cancer patients diagnosed with depression had a worse quality of life compared to cervical cancer patients without depression. A binary logistic regression analysis revealed that the following factors: no perception of external support, high VAS for pain score, high CFQ 11 fatigue score, and no previous surgical treatment for cervical cancer were all significantly higher in those cervical cancer patients diagnosed with depression (aOR = 3.12, p = 0.031; aOR = 1.25, p = 0.031; aOR = 1.35, p < 0.001; and aOR = 2.99, p = 0.035; respectively).<br>Conclusion: According to our research the prevalence of depression in Northern Thai cervical cancer patients is 13.5% and "the four factors of no perception of external support, pain, fatigue, and no previous surgical treatment for cervical cancer" associated with depression.
**Order of Authors:**	Nuntaporn Karawekpanyawong, M.D.
	Kewalee Kaewkitikul
	Benchalak Maneeton
	Narong Maneeton
	Sitthicha Siriaree
**Additional Information:**	
**Question**	**Response**
**Financial Disclosure**<br><br>Enter a financial disclosure statement that describes the sources of funding for the work included in this submission. Review the submission guidelines for detailed requirements. View published research articles from *PLOS ONE* for specific examples.	Funding for this study was supported by the Faculty of Medicine, Chiang Mai University (grant number 043/2562). The funders had no role in study design, data collection and analysis, decision to publish, or preparation of the manuscript.

This statement is required for submission and **will appear in the published article** if the submission is accepted. Please make sure it is accurate.<br><br>**Unfunded studies**<br>Enter: *The author(s) received no specific funding for this work.*<br><br>**Funded studies**<br>Enter a statement with the following details:<br>• Initials of the authors who received each award<br>• Grant numbers awarded to each author<br>• The full name of each funder<br>• URL of each funder website<br>• Did the sponsors or funders play any role in the study design, data collection and analysis, decision to publish, or preparation of the manuscript?<br>• **NO** - Include this sentence at the end of your statement: *The funders had no role in study design, data collection and analysis, decision to publish, or preparation of the manuscript.*<br>• **YES** - Specify the role(s) played.<br><br>* typeset	
**Competing Interests**<br><br>Use the instructions below to enter a competing interest statement for this submission. On behalf of all authors, disclose any competing interests that could be perceived to bias this work—acknowledging all financial support and any other relevant financial or non-financial competing interests.<br><br>This statement **will appear in the published article** if the submission is accepted. Please make sure it is accurate. View published research articles from *PLOS ONE* for specific examples.	The authors have declared that no competing interests exist.

**NO authors have competing interests**<br><br>Enter: *The authors have declared that no competing interests exist.*<br><br>**Authors with competing interests**<br><br>Enter competing interest details beginning with this statement:<br><br>*I have read the journal's policy and the authors of this manuscript have the following competing interests: [insert competing interests here]*<br><br>* typeset	
**Ethics Statement**<br><br>Enter an ethics statement for this submission. This statement is required if the study involved:<br><br>• Human participants<br>• Human specimens or tissue<br>• Vertebrate animals or cephalopods<br>• Vertebrate embryos or tissues<br>• Field research<br><br>Write "N/A" if the submission does not require an ethics statement.<br><br>General guidance is provided below. Consult the submission guidelines for detailed instructions. **Make sure that all information entered here is included in the Methods section of the manuscript.**	The Research Ethics Committee, Faculty of Medicine, Chiang Mai University approved the study protocol of the Faculty of Medicine, Chiang Mai University (reference number 399/2018). After the researcher fully explained the study details, all participants gave written informed consent before their participation.

Powered by Editorial Manager® and ProduXion Manager® from Aries Systems Corporation

**Format for specific study types**<br><br>**Human Subject Research (involving human participants and/or tissue)**<br>• Give the name of the institutional review board or ethics committee that approved the study<br>• Include the approval number and/or a statement indicating approval of this research<br>• Indicate the form of consent obtained (written/oral) or the reason that consent was not obtained (e.g. the data were analyzed anonymously)<br><br>**Animal Research (involving vertebrate animals, embryos or tissues)**<br>• Provide the name of the Institutional Animal Care and Use Committee (IACUC) or other relevant ethics board that reviewed the study protocol, and indicate whether they approved this research or granted a formal waiver of ethical approval<br>• Include an approval number if one was obtained<br>• If the study involved *non-human primates*, add *additional details* about animal welfare and steps taken to ameliorate suffering<br>• If anesthesia, euthanasia, or any kind of animal sacrifice is part of the study, include briefly which substances and/or methods were applied<br><br>**Field Research**<br><br>Include the following details if this study involves the collection of plant, animal, or other materials from a natural setting:<br>• Field permit number<br>• Name of the institution or relevant body that granted permission	
**Data Availability**<br><br>Authors are required to make all data underlying the findings described fully available, without restriction, and from the time of publication. PLOS allows rare exceptions to address legal and ethical concerns. See the PLOS Data Policy and FAQ for detailed information.	Yes - all data are fully available without restriction

A Data Availability Statement describing where the data can be found is required at submission. Your answers to this question constitute the Data Availability Statement and **will be published in the article**, if accepted.<br><br>**Important:** Stating 'data available on request from the author' is not sufficient. If your data are only available upon request, select 'No' for the first question and explain your exceptional situation in the text box.<br><br>Do the authors confirm that all data underlying the findings described in their manuscript are fully available without restriction?	
**Describe where the data may be found in full sentences. If you are copying our sample text, replace any instances of XXX with the appropriate details.**<br><br>• If the data are **held or will be held in a public repository**, include URLs, accession numbers or DOIs. If this information will only be available after acceptance, indicate this by ticking the box below. For example: *All XXX files are available from the XXX database (accession number(s) XXX, XXX.).*<br>• If the data are all contained **within the manuscript and/or Supporting Information files**, enter the following: *All relevant data are within the manuscript and its Supporting Information files.*<br>• If neither of these applies but you are able to provide **details of access elsewhere**, with or without limitations, please do so. For example:<br><br>*Data cannot be shared publicly because of [XXX]. Data are available from the XXX Institutional Data Access / Ethics Committee (contact via XXX) for researchers who meet the criteria for access to confidential data.*<br><br>*The data underlying the results presented in the study are available from (include the name of the third party*	Data cannot be shared publicly because of ethical concern. Data are available from Ethics Committee (contact author via nuntaporn.karawek@cmu.ac.th) for researchers who meet the criteria for access to confidential data.

and contact information or URL).<br>• This text is appropriate if the data are owned by a third party and authors do not have permission to share the data.<br><br>* typeset	
Additional data availability information:	

Manuscript

Click here to access/download;Manuscript;manuscript2020-10-14 for Plosone.docx

The prevalence of depression and its association in Thai cervical cancer patients

Depression in cervical cancer

Nuntaporn Karawekpanyawong[1*], Kewalee Kaewkitikul[1], Benchalak Maneeton[1], Narong Maneeton[1], Sitthicha Siriaree[2]

[1]Department of Psychiatry, Faculty of Medicine, Chiang Mai University, Chiang Mai, Thailand

[2]Department of Obstetrics and Gynecology, Faculty of Medicine, Chiang Mai University Chiang Mai, Thailand

*Corresponding author

E-mail: nuntaporn.karawek@cmu.ac.th

Abstract:

Purpose: This study examined the prevalence, risk factors, and quality of life associated with depression in cervical cancer patients.

Patients and methods: This cross-sectional, observational study was carried out in the gynecologic oncology clinic of a university hospital in Northern Thailand from October 2018 to August 2019. This study included two-hundred patients with all stages of cervical cancer. We assessed all participants with the nine-item Patient Health Questionnaire (PHQ-9; Thai version), The Diagnostic and Statistical Manual of Mental Disorders Fifth Edition (DSM-5) classification, a suicidal screening form (the 8Q), the Charlson Comorbidity Index (CCI), the eleven-item Chalder Fatigue Scale (CFQ 11), the visual analog scale (VAS) for pain, the European Organization for the Research and Treatment of Cancer Quality of Life Questionnaire Core 30 (EORTC QLQ-C30) and Cervical Cancer Module (EORTC QLQ-Cx24).

Results: Twenty-seven (13.5%) patients were diagnosed with depression by psychiatrists according to the DSM-5. The cervical cancer patients diagnosed with depression had a worse quality of life compared to cervical cancer patients without depression. A binary logistic regression analysis revealed that the following factors: no perception of external support, high VAS for pain score, high CFQ 11 fatigue score, and no previous surgical treatment for cervical cancer were all significantly higher in those cervical cancer patients diagnosed with depression (aOR = 3.12, $p = 0.031$; aOR = 1.25, $p = 0.031$; aOR = 1.35, $p < 0.001$; and aOR = 2.99, $p = 0.035$; respectively).

Conclusion: According to our research the prevalence of depression in Northern Thai cervical cancer patients is 13.5% and "the four factors of no perception of external support,

pain, fatigue, and no previous surgical treatment for cervical cancer” associated with depression.

Keywords: cervix, malignancy, depress, depressive disorder, risk, predictor, quality of life

Introduction

Cervical cancer is the fourth most common cancer among women in the world [1] and the third most common among Asian women [2]. In Thailand overall, cervical cancer was the second most common cancer. Northern Thailand has the second-highest incidence of cervical cancer, ranking after East Thailand [3].

Several cervical cancer treatment methods depend on the stage of cancer. These methods include cone biopsy or simple hysterectomy in patients with stage 1A; radical hysterectomy plus pelvic lymphadenectomy in patients with stage 1B; and primary external radiotherapy, brachytherapy, and concomitant chemoradiotherapy (CCRT) in patients with more advanced stages of cancer [4]. Since cervical cancer treatment is so effective, 5-year survival rates are high, around 70%. For this reason, quality of life becomes an important issue [5]. The goal of treating cervical cancer not only has become curing cancer itself but also improving the quality of life [6,7].

Factors associated with quality of life in cervical cancer patients have been well researched. These factors include persistent gynecologic problems, low social support, somatic symptoms, less adaptive coping, medical conditions, sleep problems, inadequate education, and depression [8–11]

Depressive disorders, major depressive disorder, and persistent depressive disorder, cause significantly marked sadness and affect feelings, thought, and behavior. Depressive disorders can lead to a variety of emotional and physical problems [12]. Symptoms of depression can increase mortality, decrease adherence to medication, and decrease the quality of life in cervical cancer patients [13,14]. In the general population, depressive disorder is the highest prevalence of any psychiatric disorder, at about 5-17% worldwide, but only 0.3-2.4% in Thailand [15,16].

In contrast, the prevalence of depression among cervical cancer patients was higher. Not only can the disease cause depression in several ways, due to its physical, emotional, and psychological effects but the treatment may also lead to depression, due to the involvement of reproductive and hormonal systems, thereby affecting the identity, self-image, and self-esteem [17–20].

The prevalence of depression in cervical cancer patients has been vastly researched but the results have been [illegible]rious, 4.21% to 80%, depending on the measures of depression and culture of the participants [18–28]. In Thailand, only two studies examined the prevalence of depression and its association among gynecol[illegible]c cancer but no study mentioned cervical cancer [17,29].

So, the objective of this study was to investigate the prevalence of depression in cervical cancer patients in Northern Thailand. Also, the study examined risk factors associated with depression to determine effective management in these patients.

Material and methods

Aims and objectives

1. Primary objective

To determine the prevalence of depression in patients with cervical cancer in Northern Thailand

2. Secondary objectives

To determine the effect of depression on the quality of life in this population.

To determine the risk factors for depression in cervical cancer patients in this population.

Method

This study was a cross-sectional, observational study in patients receiving services at the gynecologic oncology clinic of a university hospital in Northern Thailand. The patients completed all questionnaires in a single visit. The Research Ethics Committee, Faculty of Medicine, Chiang Mai University approved the study protocol of the Faculty of Medicine, Chiang Mai University (reference number 399/2018). After the researcher fully explained the study details, all participants gave written informed consent before their participation.

Participants

We invited all cervical cancer patients who visited the gynecologic oncology clinic to participate in the study. Exclusion criteria were the inability to read/write Thai and significant cognitive impairment.

Sample size

Sample size was calculated using "$N= Z^2 (PQ) / E^2$", where Z= 1.96 (95% confidence level). P is the percentage of the study phenomena from a previous study. For our study, P=0.078, the prevalence of depression in cervical cancer from the most recent study in Asia [25]. Q is 1-P, or 0.922. E is the accepted bias for p in the sample, that is 0.05. Calculated sample size was N=110; 200 patients were interviewed according to the assumption that half of patients might not complete the interview due to fatigue.

Assessment

Cervical cancer, Medical Illnesses, Pain and Fatigue

We recorded demographic data: age, year of education, marital status, support systems, personal income, and financial problems from a questionnaire. We reviewed the duration of cancer, stage of cancer, and treatment regimens from medical records.

We assessed 16 physical illnesses affecting mortality rates by using the Charlson Comorbidity Index (CCI) [30]. Comorbid diseases with a score of 1 included myocardial infarction,

congestive heart failure, peripheral vascular disease, cerebrovascular disease, deme[illegible], chronic obstructive pulmonary disease, connective tissue disease, peptic ulcer disease, uncomplicated diabetes mellitus, and mild liver disease. More serious illnesses including diabetes mellitus with end□ organ damage, moderate to severe chronic kidney disease, hemiplegia, leukemia, malignant lymphoma, and solid tumor applied a score of 2. Moderate to severe liver disease scored 3. Metastatic cancer and Acquired Immune Deficiency Syndrome scored 6. We use the visual analog scale (VAS) for evaluating the pain and the eleven-item Chalder Fatigue Scale (CFQ 11) for evaluating fatigue [31].

Depression

The participants were divided into two groups, depression [illegible] non-depression. We screened depression by using a self-rating questionnaire, the nine-item Patient Health Questionnaire (PHQ-9; Thai version) [32–34]. This questionnaire asked the frequency of feeling relevant to depressive symptoms in the past two weeks. Each question scored from 0 (not at all) to 3 (nearly every day). The recommended cut-off point for depression was nine or greater. The sensitivity at this cut-off value was 0.84, and the specificity was 0.77. Besides, we assessed patients who had positive screening from the PHQ-9; Thai version by psychiatr[illegible] according to The Diagnostic and Statistical Manual of Mental Disorders, Fifth Edition (DSM-5) classification [12]. The depression group included participants who were diagnosed with any depressive disorder at the study time.

Quality of life

We assessed the quality of life by using the European Organization for the Research and Treatment of Cancer Quality of Life Questionnaire Core 30 (EORTC QLQ-C30) and Cervical Cancer Module (EORTC QLQ-Cx24). The EORTC Quality of Life Group provided this instrument. We have obtained permission to use the instruments from the institutions.

The EORTC QLQ-C30 assesses the activity limitations experienced in cancer patients. Its 30 items include: a 2-item global health status or quality of life scale; 28-item functional difficulty, or symptom scales. The levels of problems and symptoms ranged from 1 (not at all) to 4 (very much). The final scores ranged from 28 (no disability) to 112 (the most severe disability). The reliability of the Thai-version of this tool using internal consistency, with Cronbach's alpha, was 0.7 [35,36].

The EORTC QLQ-Cx24 was a 24-item questionnaire to assess patients' perception of symptoms related to cervical cancer. It consists of four functioning domains: body image, sexual activity, sexual enjoyment, and sexual/vaginal functioning; and five symptoms domain: symptoms experienced, lymphoedema, peripheral neuropathy, menopausal symptoms, and sexual worry. The higher scores of all areas except sexual activity and sexual enjoyment refer to the low quality of life-related to cervical cancer. The reliability of the Thai-version EORTC QLQ-Cx24 using internal consistency, with Cronbach's alpha equal to 0.79 [37].

Statistical Analysis

Participants and Prevalence

All sociodemographic, clinical characteristics, and quality of life were assessed between the two g[illegible]ps and presented the frequency and percentage for categorical data. We presented mean with standard deviation or median with interquartile range for continuous data depending on the distribution.

Depression Risk Factors

Univariable logistic regression was used for potential risk [illegible]ors of depression among cervical cancer patients. The significant factors in univariable analyses associated with depression ($p < 0.2$ to enter) were included in the binary logistic regression model. We presented the association as odds ratios (ORs) with 95% confidence intervals (CIs).

All analyses used SPSS version 24.00.

Results

Participants and prevalence

This study was carried out in the gynecologic oncology clinic of a university hospital in Northern Thailand from October 2018 to August 2019. A total of 200 patients visited the clinic during the study enrollment. The median age and median duration of illness were 56 years (IQR = 49-62) and 22 months (IQR = 6-34), respectively. Thirty-one patients (15.5%) had positive screening from PHQ-9, but only twenty-seven patients (13.5%) received a diagnosis of depressive disorder. The twenty-six patients (13%) had a major depressive disorder, and one patient had a persistent depressive disorder with intermittent major depressive episode without current episode (0.5%) according to DSM-5 classification. This study found that thirteen patients (6.5%) had a suicidal idea, but only one patient had high-risk suicidality (0.5%).

Comparison of quality of life between the depression and the nondepression group

The depression group had significantly lower means of EORTC QLQ-C30 functioning scores in all items. The means of EORTC QLQ-C30 symptoms scores in the depression group were higher than the nondepression group significantly except for constipation and diarrhea. The means of symptoms experience score, body image score, and sexual worry score from EORTC QLQ-Cx24 in the depression group were significantly higher. Likewise, the depression group had significantly lower sexual activity. The results showed depression in cervical cancer patients correlated with worse quality of life in almost all domains, as shown in Figure 1-3.

Figure 1. EORTC QLQ-C30 functioning mean scores of the depression group compared to the nondepression group, with the p-value. A higher percentage on the global quality of life, functioning scales indicate a better quality of life. EORTC QLQ-C30, European Organisation for Research and Treatment of Cancer Quality of Life Core Questionnaire.

Figure 2. EORTC QLQ-C30 symptoms mean scores of the depression group compared to the nondepression group, with the p-value. A higher percentage on symptom scales indicates a poorer quality of life. EORTC QLQ-C30, European Organisation for Research and Treatment of Cancer Quality of Life Core Questionnaire.

Figure 3. EORTC QLQ- Cx24 mean scores of the depression group compared to the nondepression group, with the p-value. A higher percentage on cervical-related symptom scales indicates a poorer quality of life. A higher percentage on sexual activity, and sexual enjoyment indicate a better quality of life. EORTC QLQ-Cx24, EORTC Cervical Cancer Module.

Binary Logistic Regression for Depression Risk Factors

This study analyzed all of the factors associated with depression according to the literature review, as shown in Table 1. From the univariable analysis, twelve factors were significantly ($p > 0.2$) associated with depression in cervical cancer patients, including age, no perception of external support, financial problems, menopausal symptoms, high pain score, high fatigue score, long duration of cancer, advance stage of cancer, no previous surgery, previous chemotherapy, current chemotherapy, and stage of treatment

Table 1 Univariable Logistic Regression for Potential Risk Factors of Depression Among Cervical Cancer Patients

Characteristics,<br>n (%) / Median [IQR]		All<br>(n = 200)	Depression<br>(n = 27)	Non-depression<br>(n = 173)	OR (95% CI)	p-value
Age (years)		56 [49-62]	51 [48-58]	57 [50-62]	0.97 (0.93-1.01)	0.148
Educational level						0.452
	No	28 (14.0%)	6 (22.2%)	22 (12.7%)	1.00	
	Primary school	123 (61.5%)	15 (55.6%)	108 (62.4%)	0.51 (0.18-1.46)	
	Secondary school	23 (11.5%)	4 (14.8%)	19 (10.9%)	0.77 (0.19-3.15)	
	Higher education	26 (13.0%)	2 (7.4%)	24 (13.9%)	0.31 (0.06-1.68)	
Marital status						0.934
	Single	8 (4.0%)	1 (3.7%)	7 (4.0%)	1.00	
	Married	146 (73.0%)	21 (77.8%)	125 (72.3%)	1.18 (0.14-10.05)	
	Separated or divorced	21 (10.5%)	2 (7.4%)	19 (11.0%)	0.74 (0.06-9.46)	
	Widowed	25 (12.5%)	3 (11.1%)	22 (12.7%)	0.96 (0.09-10.71)	
Number of children						0.954
	No child	17 (8.5%)	2 (7.4%)	15 (8.7%)	1.00	
	1 child	48 (24.0%)	7 (25.9%)	41 (23.7%)	1.28 (0.24-6.86)	
	≥2 children	135 (67.5)	18 (66.7%)	117 (67.6%)	1.15 (0.24-5.47)	
Perception of external support		149 (74.5%)	16 (59.3%)	133 (76.9%)	0.44 (0.19-1.02)	0.055
Have family history of psychiatric disorder		11 (5.5%)	1 (3.7%)	10 (5.8%)	0.63 (0.08-5.10)	0.663
Personal income per month						0.756
	>15000 bath	23 (11.5%)	2 (7.4%)	21 (12.1%)	1.00	

10001-15000 bath	15 (7.5%)	0 (0%)	15 (8.7%)	NA	
5001-10000 bath	27 (13.5%)	3 (11.1%)	24 (13.9%)	1.31 (0.20-8.62)	
<5000 bath	135 (67.5%)	22 (81.5%)	113 (65.3%)	2.04 (0.45-9.35)	
Financial problem					0.056
No	83 (41.5%)	5 (18.5%)	78 (45.1%)	1.00	
Low	49 (24.5%)	7 (25.9%)	42 (24.3%)	2.60 (0.78-8.70)	
Moderate	34 (17.0%)	7 (25.9%)	27 (15.6%)	4.04 (1.18-13.81)	
Severe	34 (17.0%)	8 (29.6%)	26 (15.0%)	4.80 (1.44-15.97)	
Not menopause	173 (86.5%)	22 (81.5%)	151 (87.3%)	1.56 (0.54-4.54)	0.415
Menopausal symptoms	91 (45.5%)	17 (63.0%)	74 (42.8%)	2.27 (0.99-5.25)	0.054
CCI score	4 [3-5]	3 [2-6]	4 [3-5]	1.02 (0.86-1.21)	0.812
VAS for pain score	2 [0-5]	5 [3-5]	2 [0-5]	1.34 (1.13-1.58)	0.001
CFQ 11 fatigue score	16 [13-20]	21 [18-23]	16 [13-19]	1.38 (1.21-1.56)	<0.001
Duration of cancer	22 [3-64]	10 [2-37]	23 [3-71]	0.99 (0.99-1.00)	0.102
Type of cancer					0.510
Squamous cell	145 (72.5%)	21 (77.8%)	124 (71.7%)	1.00	
Non-squamous cell	55 (27.5%)	6 (22.2%)	49 (28.3%)	0.72 (0.28-1.90)	
Stage of cancer					0.049
Stage 1	90 (45.0%)	6 (22.2%)	84 (48.6%)	1.00	
Stage 2	67 (33.5%)	13 (48.1%)	54 (31.2%)	3.37 (1.21-9.40)	
Stage 3,4	43 (21.5%)	8 (29.7%)	35 (20.2%)	3.20 (1.03-9.90)	
Recurrent of cancer	16 (8.0%)	2 (7.4%)	14 (8.1%)	0.91 (0.20-4.24)	0.903

Metastasis of cancer	3 (1.5%)	1 (3.7%)	2 (1.2%)	3.29 (0.29-37.57)	0.338
Previous surgery	120 (60.0%)	11 (40.7%)	109 (63.0%)	0.40 (0.18-0.92)	0.032
Previous RT	149 (74.5%)	22 (81.5%)	127 (73.4%)	1.59 (0.57-4.46)	0.374
Previous chemotherapy	134 (67.0%)	22 (81.5%)	112 (64.7%)	2.40 (0.86-6.65)	0.093
Previous hormones	8 (4%)	1 (3.7%)	7 (4.0%)	0.91 (0.11-7.72)	0.933
Previous brachytherapy	59 (29.5%)	9 (33.3%)	50 (28.9%)	1.23 (0.52-2.92)	0.639
Current chemotherapy	71 (35.5%)	14 (51.9%)	57 (32.9%)	2.19 (0.97-4.97)	0.060
Stage of treatment					0.042
Annual check up	108 (54.0%)	9 (33.4%)	99 (57.2%)	1.00	
First line	70 (35.0%)	12 (44.4%)	58 (33.5%)	2.28 (0.90-5.73)	
Second or Third line	22 (11.0%)	6 (22.2%)	16 (9.3%)	4.13 (1.29-13.16)	

Abbreviations: IQR, Interquartile ranges; OR, odd ratio; CI, confidence interval; CCI, the Charlson Comorbidity Index; CFQ 11, the eleven-item Chalder Fatigue Scale; VAS, the visual analog scale

After adjusting for those significant factors, only four factors were significantly associated with depression in cervical cancer patients, including perceived no social support, higher pain score, higher fatigue score, and no previous surgery (aOR =3.12, p = 0.031, aOR =1.25, p = 0.031, aOR = 1.35, p < 0.001, aOR = 2.99, p = 0.035 respectively), as shown in Table 2.

Table 2 Multivariable Logistic Regression for Independent Risk Factors of Depression Among Cervical Cancer Patients

**Characteristics**	**PHQ-9 (cut-point 10)**	
	**aOR (95% CI)**	**p-value**

No perception of external support	3.12 (1.11-8.81)	0.031
VAS for pain score	1.25 (1.02-1.54)	0.031
CFQ 11 fatigue score	1.35 (1.18-1.53)	<0.001
No previous surgery	2.99 (1.08-8.29)	0.035

Note: Variables with p-value <0.20 in univariable analysis were included in multivariable analysis

Abbreviations: aOR, adjusted odd ratio; CI, confidence interval; CCI, the Charlson Comorbidity Index; CFQ 11, the eleven-item Chalder Fatigue Scale; VAS, the visual analog scale

Discussion

Prevalence of depression in cervical cancer

From our study, the prevalence of depression in cervical cancer patients in Northern Thailand was 13.5%. This prevalence is five times higher than the general population of Thailand [15]. In contrast with one study in Korea, the prevalence of depression in cervical cancer patients was less than in the healthy control group [22].

The present study demonstrated that this prevalence is low compared with several studies around the world. Although, there are ample studies that examined the prevalence of depression in cervical cancer patients the results varied from 4.21% to 80%. A key difference among those studies were the measures used for identifying depression. Most studies measured depression using self-report screening tools such as the Hospital Anxiety and Depression Scale [18,21,22,28], the Beck Depression Inventory [26], the Center for Epidemiologic Studies -

Depression Scale [20], and the health-related self-report [17]. Some studies measured depression using structural clinical interviews such as the Chinese-Bilingual Structured Clinical Interview [24] and the Mini-International Neuropsychiatric Interview [29]. Two other studies defined depression from the database system be compatible with the International Classification of Diseases code [25,27]. To our knowledge, this is the first study using the diagnosis of psychiatrists according to DSM-5 to detect depression in cervical cancer patients.

The present 13.5% prevalence rate of depression is lower than those 21.3-80% prevalence rate from studies that defined depression using the screening tool or the structural clinical intervi[illegible] That higher prevalence from screening or structural clinical interview can be attributed to the overlap between the symptoms of cancer or treatment side effects and the symptoms of depression. Symptoms including loss of appetite, fatigue, sleep difficulty are very common side effects of chemotherapy and radiotherapy treatment.

In contrast, this present prevalence is higher than those 4.21-7.8% prevalence rate from studies that defined depression by using diagnosis from the ICD code data[illegible]e. The lower prevalence by using diagnosis from the ICD code database can be attributed to the weakness of the secondary data. No evidence confirms that all patients were evaluated for depression.

So, we have two ideas from this result. First, researchers should estimate the size of this problem not only by considering the culture but also by the type of measurement.
Second, this result confirmed that there was more prevalence of depression in this population so regular screening for depressive disorder in those patients is beneficial.

Quality of life and depression

This study showed that depression correlated with quality of life similar to results from many prior studies [9,11,19,23].

This study found that cervical cancer patients diagnosed with depressive disorder have a lower function but higher cancer-related symptoms both general cancer-related symptoms

and cervical cancer-related symptoms. Those patients report more concern about their body image and pain during sexual activity. Not surprisingly, this group of patients has less sexual activity.

Since depressive disorder affects the quality of life in this population, optimal treatment for depression is beneficial.

Factors associated with depression

The present result revealed that four factors correlated with depression in cervical cancer patients.

The first factor was no perception of social support. Similar to previous studies, no perception of social support increases the risk of depression in gynecologic cancer patients including cervical cancer [22,38–40]. Several studies imparted the close relationship between social support and depression. Those revealed that social support influences vulnerabilities and resilience to stress from both biological and psychological ways. Biological impacts affect the hypothalamic-pituitary-adrenocortical (HPA) system, the noradrenergic system, and central oxytocin pathways. The psychological impact is hope [41]. A study showed cancer patients need social support more than the general population [42,43]. Therefore, encouraging social support helps prevent and alleviate depression, especially supporting from their spouses, family, and friends [42,44,45].

The second factor was the pain. Ample studies found a close relationship between pain and depression including in patients with cervical cancer [20,21,24,29,46–48]. The interaction has been labeled as the depression-pain syndrome or depression-pain dyad, implying that the conditions often coexist, respond to similar treatments, exacerbate one another, and share biological pathways and neurotransmitters. Both depression and pain are caused by dysfunctions of serotonin and norepinephrine neurotransmitters. Moreover, depression impacts pain modulation via psychological mechanisms involving patient's

expectations, increasing attention, and amplifying negative emotional reactions to pain [49]. Since pain is associated with depression, adequate pain control is beneficial in this population.

The third factor was fatigue. The prevalence of fatigue in cervical cancer patients was 23% and higher in metastasis and during chemoradiation treatment [50]. Conversely, the prevalence in the general population was only 10% [51]. A previous study reported that cancer-related fatigue might reduce the quality of life and cause a mental illness especially depression in these patients [52,53,53]. Although fatigue often coexists with depression, it may also cause depression. Little has been known about the link between fatigue and depression in cervical cancer patients. However, such a correlation may be explained by the biopsychosocial model. The psychological model, fatigue might provide difficulty in daily activity that triggered a sense of low self-esteem and depression. The biological model, fatigue shares biological dysfunctions with depression. Biological dysfunctions are serotonin (5-HT) dysregulation, hypothalamic-pituitary-adrenal (HPA) axis dysfunction, circadian rhythm disruption, vagal afferent nerve activation, and cytokine dysregulation [54,55]. For this reason, screening for depression in cervical cancer patients who have fatigue is beneficial [56].

Finally was no previous surgical treatment for cancer. This finding is contrary to previous studies which have suggested that cervical cancer patients who underwent surgery experienced the highest rates of depressive symptoms compared with other treatment methods [57]. The present result may partly be explained by culture and fear. A study from Thailand found that one of the greatest fears in Thai cervical cancer patients was fear of disease progression and those fears correlated with a higher score of depression [58]. Cervical cancer patients who underwent surgical treatment may have less fear because they think that they have already eliminated the primary source of cancer. Another study in Thailand supported this hypothesis because it found that cervical cancer survivors treated with radical

hysterectomy may have a better quality of life than healthy peers [59]. Since this result, reassurance about treatment and improvement of the monitoring program may have an advantage.

Limitations

The present study had several limitations. First, we used a convenient sample from Northern Thailand, which limited the generalizability of the findings to other cervical cancer patients or the different cultural contexts. Further studies need to be conducted to examine whether the results of the present study are suitable for different cultural contexts. Second, this study was a cross- sectional design, which cannot explain the causal relationship between factors and depression. Further longitudinal studies are needed to validate the current findings. Third, the interpretation of the results should be made with caution because we did not include a control group. If comparable control groups are involved, the level of depression in cancer patients can be reliably and accurately determined. Finally, data were mainly obtained using self-administered questions, so there was a possibility of recall and reporting bias.

Conclusion

Our study showed that the prevalence of a depressive disorder in cervical cancer patients in Northern Thailand was rather low compared with other countries, only 13.5%. This study confirmed the association between depression and quality of life. Four factors associated with depression were no perception of social support, pain, fatigue, and no previous surgical treatment for cancer. These results suggest that regular screening for depression and improvement of the intervention for patients who have those risks may helpfully to decrease the number of depression and also improve the quality of life in cervical cancer patients.

Acknowledgments

Funding for this study was supported by the Faculty of Medicine, Chiang Mai University. We wish to thank Ms. Ruth Leatherman for her assistance in manuscript English-language editing. We are grateful to Professor Manit Srisurapanont and Mr. Suttipong Kawilapat, Department of Psychiatry, Faculty of Medicine, Chiang Mai University, who provided statistical advice.

Disclosure

The authors report no conflicts of interest in this work.

References

1. Aaronson, N. K., Ahmedzai, S., Bergman, B., Bullinger, M., Cull, A., Duez, N. J., Filiberti, A., Flechtner, H., Fleishman, S. B., Haes, J. C. J. M. de, Kaasa, S., Klee, M., Osoba, D., Razavi, D., Rofe, P. B., Schraub, S., Sneeuw, K., Sullivan, M., & Takeda, F. (1993). The European Organization for Research and Treatment of Cancer QLQ-C30: A Quality-of-Life Instrument for Use in International Clinical Trials in Oncology. *JNCI: Journal of the National Cancer Institute, 85*(5), 365–376. https://doi.org/10.1093/jnci/85.5.365
2. Aoki, E. S., Yin, R., Li, K., Bhatla, N., Singhal, S., Ocviyanti, D., Saika, K., Suh, M., Kim, M., & Termrungruanglert, W. (2020). National screening programs for cervical cancer in Asian countries. *Journal of Gynecologic Oncology, 31*(3). https://doi.org/10.3802/jgo.2020.31.e55
3. Arbyn, M., Weiderpass, E., Bruni, L., Sanjosé, S. de, Saraiya, M., Ferlay, J., & Bray, F. (2020). Estimates of incidence and mortality of cervical cancer in 2018: A worldwide

analysis. *The Lancet Global Health*, *8*(2), e191–e203. https://doi.org/10.1016/S2214-109X(19)30482-6

4. Association, A. P. (2013). *Diagnostic and Statistical Manual of Mental Disorders (DSM-5®)*. American Psychiatric Publishing. https://books.google.co.th/books?id=-JivBAAAQBAJ
5. Bair, M. J., Robinson, R. L., Katon, W., & Kroenke, K. (2003). Depression and Pain Comorbidity: A Literature Review. *Archives of Internal Medicine*, *163*(20), 2433–2445. https://doi.org/10.1001/archinte.163.20.2433
6. Bower, J. E. (2014). Cancer-related fatigue: Mechanisms, risk factors, and treatments. *Nature Reviews. Clinical Oncology*, *11*(10), 597–609. https://doi.org/10.1038/nrclinonc.2014.127
7. Carpenter, K. M., Fowler, J. M., Maxwell, G. L., & Andersen, B. L. (2010). Direct and Buffering Effects of Social Support among Gynecologic Cancer Survivors. *Annals of Behavioral Medicine : A Publication of the Society of Behavioral Medicine*, *39*(1), 79–90. https://doi.org/10.1007/s12160-010-9160-1
8. Charlson, M. E., Pompei, P., Ales, K. L., & MacKenzie, C. R. (1987). A new method of classifying prognostic comorbidity in longitudinal studies: Development and validation. *Journal of Clinical Epidemiology*, *40*(5), 373–383.
9. Daniëls, L. A., Oerlemans, S., Krol, A. D. G., van de Poll-Franse, L. V., & Creutzberg, C. L. (2013). Persisting fatigue in Hodgkin lymphoma survivors: A systematic review. *Annals of Hematology*, *92*(8), 1023–1032. https://doi.org/10.1007/s00277-013-1793-2
10. Ebede, C. C., Jang, Y., & Escalante, C. P. (2017). Cancer-related fatigue in cancer survivorship. *Medical Clinics*, *101*(6), 1085–1097.
11. Gariépy, G., Honkaniemi, H., & Quesnel-Vallée, A. (2016). Social support and protection from depression: Systematic review of current findings in Western countries. *The British*

Journal of Psychiatry: The Journal of Mental Science, *209*(4), 284–293. https://doi.org/10.1192/bjp.bp.115.169094

12. Greimel, E. R., Kuljanic Vlasic, K., Waldenstrom, A., Duric, V. M., Jensen, P. T., Singer, S., Chie, W., Nordin, A., Bjelic Radisic, V., & Wydra, D. (2006). The European Organization for Research and Treatment of Cancer (EORTC) Quality□ of□ Life questionnaire cervical cancer module: EORTC QLQ□CX24. *Cancer, 107*(8), 1812–1822.
13. Hanprasertpong, J., Geater, A., Jiamset, I., Padungkul, L., Hirunkajonpan, P., & Songhong, N. (2017). Fear of cancer recurrence and its predictors among cervical cancer survivors. *Journal of Gynecologic Oncology, 28*(6), e72. https://doi.org/10.3802/jgo.2017.28.e72
14. Hengrasmee, P., Padungsutt, P., & Boriboonhirunsarn, D. (2004). *Depression among Gynecologic Cancer Patients at Siriraj Hospital: Prevalence and Associated Factors*. *87*, 6.
15. Hong, J. S., & Tian, J. (2014). Prevalence of anxiety and depression and their risk factors in Chinese cancer patients. *Supportive Care in Cancer*, *22*(2), 453–459. https://doi.org/10.1007/s00520-013-1997-y
16. Imsamran, W., Pattatang, A., Supaattagorn, P., Chiawiriyabunya, I., Namthaisong, K., & Suwanrungruang, K. (2018). *Cancer in Thailand: Vol. IX, 2013-2015*. Bangkok: New Thammada Press.
17. Jyani, G., Chauhan, A. S., Rai, B., Ghoshal, S., Srinivasan, R., & Prinja, S. (2020). Health-related quality of life among cervical cancer patients in India. *International Journal of Gynecological Cancer: Official Journal of the International Gynecological Cancer Society*. https://doi.org/10.1136/ijgc-2020-001455
18. Kim, S. H., Kang, S., Kim, Y.-M., Kim, B.-G., Seong, S. J., Cha, S. D., Park, C.-Y., & Yun, Y. H. (2010). Prevalence and predictors of anxiety and depression among cervical cancer survivors in Korea. *International Journal of Gynecological Cancer: Official*

Journal of the International Gynecological Cancer Society, *20*(6), 1017–1024. https://doi.org/10.1111/IGC.0b013e3181e4a704

19. Kim, S., Nam, J., Park, S., Bae, D., Park, C., Cho, C., Lee, J., & Yun, Y. (2009). Study of anxiety and depression in cervical cancer survivors. *Journal of Clinical Oncology*, *27*(15_suppl), e20644–e20644. https://doi.org/10.1200/jco.2009.27.15_suppl.e20644
20. Klapheke, A. K., Keegan, T. H. M., Ruskin, R., & Cress, R. D. (2020). Depressive symptoms and health-related quality of life in older women with gynecologic Cancers. *Journal of Geriatric Oncology*, *11*(5), 820–827. https://doi.org/10.1016/j.jgo.2019.10.001
21. Klügel, S., Lücke, C., Meta, A., Schild-Suhren, M., Malik, E., Philipsen, A., & Müller, H. H. (2017). Concomitant psychiatric symptoms and impaired quality of life in women with cervical cancer: A critical review. *International Journal of Women's Health*, *9*, 795–805. https://doi.org/10.2147/IJWH.S143368
22. Koh, W.-J., Abu-Rustum, N. R., Bean, S., Bradley, K., Campos, S. M., Cho, K. R., Chon, H. S., Chu, C., Clark, R., Cohn, D., Crispens, M. A., Damast, S., Dorigo, O., Eifel, P. J., Fisher, C. M., Frederick, P., Gaffney, D. K., Han, E., Huh, W. K., … Scavone, J. L. (2019). Cervical Cancer, Version 3.2019, NCCN Clinical Practice Guidelines in Oncology. *Journal of the National Comprehensive Cancer Network: JNCCN*, *17*(1), 64–84. https://doi.org/10.6004/jnccn.2019.0001
23. Kongsuk, T., Kittirattanapaiboon, P., Kenbubpha, K., Sukawaha, S., & Leejongpermpoon, J. (2010). The prevalence of major depressive disorders in Thailand: Results from the Epidemiology of Mental Disorders National Survey 2008. *World Psychiatric Association Section on Epidemiology and Public Health Meeting, Prediction in Psychiatric Epidemiology–from Childhood and Adolescence to Adulthood*, 11–14.

24. Korff, M. V., & Simon, G. (1996). The Relationship Between Pain and Depression. *The British Journal of Psychiatry*, *168*(S30), 101–108. https://doi.org/10.1192/S0007125000298474
25. Kroenke, K., Spitzer, R. L., & Williams, J. B. (2001). The PHQ-9: Validity of a brief depression severity measure. *Journal of General Internal Medicine*, *16*(9), 606–613.
26. Lau, K. L., Yim, P. H. W., & Cheung, E. Y. W. (2013). Psychiatric morbidity in Chinese women after cervical cancer treatment in a regional gynaecology clinic. *East Asian Archives of Psychiatry: Official Journal of the Hong Kong College of Psychiatrists = Dong Ya Jing Shen Ke Xue Zhi: Xianggang Jing Shen Ke Yi Xue Yuan Qi Kan*, *23*(4), 144–153.
27. Lotrakul, M., Sumrithe, S., & Saipanish, R. (2008). Reliability and validity of the Thai version of the PHQ-9. *BMC Psychiatry*, *8*(1), 46.
28. Lu, D., Andrae, B., Valdimarsdóttir, U., Sundström, K., Fall, K., Sparén, P., & Fang, F. (2019). Psychologic Distress Is Associated with Cancer-Specific Mortality among Patients with Cervical Cancer. *Cancer Research*, *79*(15), 3965–3972. https://doi.org/10.1158/0008-5472.CAN-19-0116
29. Manea, L., Gilbody, S., & McMillan, D. (2015). A diagnostic meta-analysis of the Patient Health Questionnaire-9 (PHQ-9) algorithm scoring method as a screen for depression. *General Hospital Psychiatry*, *37*(1), 67–75.
30. Maneeton, B., Maneeton, N., & Mahathep, P. (2012). Prevalence of depression and its correlations: A cross-sectional study in Thai cancer patients. *Asian Pacific Journal of Cancer Prevention: APJCP*, *13*(5), 2039–2043. https://doi.org/10.7314/apjcp.2012.13.5.2039
31. Maneeton, B., Maneeton, N., Reungyos, J., Intaprasert, S., Leelarphat, S., & Thongprasert, S. (2014). Prevalence and relationship between major depressive disorder and lung cancer:

A cross- sectional study. *OncoTargets and Therapy*, *7*, 815– 821. https://doi.org/10.2147/OTT.S60000

32. Manne, S. L., Kashy, D. A., Virtue, S., Criswell, K. R., Kissane, D. W., Ozga, M., Heckman, C. J., Stapleton, J., & Rodriguez, L. (2018). Acceptance, social support, benefit-finding, and depression in women with gynecological cancer. *Quality of Life Research: An International Journal of Quality of Life Aspects of Treatment, Care and Rehabilitation*, *27*(11), 2991–3002. https://doi.org/10.1007/s11136-018-1953-x
33. Mccorkle, R., Tang, S. T., Greenwald, H., Holcombe, G., & Lavery, M. (2006). Factors Related to Depressive Symptoms Among Long-Term Survivors of Cervical Cancer. *Health Care for Women International*, *27*(1), 45–58. https://doi.org/10.1080/07399330500377507
34. Meyerowitz, B. E., Formenti, S. C., Ell, K. O., & Leedham, B. (2000). Depression Among Latina Cervical Cancer Patients. *Journal of Social and Clinical Psychology*, *19*(3), 352–371. https://doi.org/10.1521/jscp.2000.19.3.352
35. Morriss, R., Wearden, A., & Mullis, R. (1998). Exploring the validity of the Chalder Fatigue scale in chronic fatigue syndrome. *Journal of Psychosomatic Research*, *45*(5), 411–417.
36. National Cancer Institute. (2016). *SEER cancer statistics review 1975-2016.*
37. Nuhu, F. T., Odejide, O. A., Adebayo, K. O., & Yusuf, A. J. (2009). Psychological and physical effects of pain on cancer patients in Ibadan, Nigeria. *African Journal of Psychiatry*, *12*(1), 64–70. https://doi.org/10.4314/ajpsy.v12i1.30281
38. Osann, K., Hsieh, S., Nelson, E. L., Monk, B. J., Chase, D., Cella, D., & Wenzel, L. (2014). Factors associated with poor quality of life among cervical cancer survivors: Implications for clinical care and clinical trials. *Gynecologic Oncology*, *135*(2), 266–272. https://doi.org/10.1016/j.ygyno.2014.08.036

39. Ozbay, F., Johnson, D. C., Dimoulas, E., Morgan, C. A., Charney, D., & Southwick, S. (2007). Social Support and Resilience to Stress. *Psychiatry (Edgmont)*, *4*(5), 35–40.

40. Park, B., Youn, S., Yi, K.-K., Lee, S., Lee, J.-S., & Chung, S. (2017). The Prevalence of Depression among Patients with the Top Ten Most Common Cancers in South Korea. *Psychiatry Investigation*, *14*(5), 618–625. https://doi.org/10.4306/pi.2017.14.5.618

41. Paul, R., Musa, G., & Chungu, H. (2016). Prevalence of Depression among Cervical Cancer Patients Seeking Treatment at the Cancer Diseases Hospital. *IOSR J Dent Med Sci Ver XI*, *15*(6), 2279–2861.

42. Petersen, R. W., Graham, G., & Quinlivan, J. A. (2005). Psychologic changes after a gynecologic cancer. *Journal of Obstetrics and Gynaecology Research*, *31*(2), 152–157. https://doi.org/10.1111/j.1341-8076.2005.00263.x

43. Pinquart, M., & Duberstein, P. R. (2010). Depression and cancer mortality: A meta-analysis. *Psychological Medicine*, *40*(11), 1797–1810. https://doi.org/10.1017/S0033291709992285

44. Prasongvej, P., Nanthakomon, T., Jaisin, K., Chanthasenanont, A., Lertvutivivat, S., Tanprasertkul, C., Bhamarapravatana, K., & Suwannarurk, K. (2017). Quality of Life in Cervical Cancer Survivors and Healthy Women: Thai Urban Population Study. *Asian Pacific Journal of Cancer Prevention : APJCP*, *18*(2), 385–389. https://doi.org/10.22034/APJCP.2017.18.2.385

45. Sadock, B. J., Sadock, V. A., & Ruiz, P. (2015). *Kaplan & Sadock's Synopsis of Psychiatry: Behavioral Sciences/ clinical Psychiatry*. Wolters Kluwer. https://books.google.co.th/books?id=hDyNnQEACAAJ

46. Shirali, E., Yarandi, F., Ghaemi, M., & Montazeri, A. (2020). Quality of Life in Patients with Gynecological Cancers: A Web-Based Study. *Asian Pacific Journal of Cancer Prevention*, *21*(7), 1969–1975. https://doi.org/10.31557/APJCP.2020.21.7.1969

47. Shyu, I.-L., Hu, L.-Y., Chen, Y.-J., Wang, P.-H., & Huang, B.-S. (2019). Risk factors for developing depression in women with cervical cancer: A nationwide population-based study in Taiwan. *International Journal of Women's Health*, *11*, 135–141. https://doi.org/10.2147/IJWH.S193003

48. Silpakit, C., Sirilerttrakul, S., Jirajarus, M., Sirisinha, T., Sirachainan, E., & Ratanatharathorn, V. (2006). The European Organization for Research and Treatment of Cancer Quality of Life Questionnaire (EORTC QLQ-C30): Validation Study of the Thai Version. *Quality of Life Research*, *15*(1), 167–172. https://doi.org/10.1007/s11136-005-0449-7

49. Singh, U., Verma, M. L., Rahman, Z., Qureshi, S., & Srivastava, K. (2019). Factors affecting quality of life of cervical cancer patients: A multivariate analysis. *Journal of Cancer Research and Therapeutics*, *15*(6), 1338.

50. Steen, R., Dahl, A. A., Hess, S. L., & Kiserud, C. E. (2017). A study of chronic fatigue in Norwegian cervical cancer survivors. *Gynecologic Oncology*, *146*(3), 630–635. https://doi.org/10.1016/j.ygyno.2017.05.028

51. Traeger, L., Braun, I. M., Greer, J. A., Temel, J. S., Cashavelly, B., & Pirl, W. F. (2011). Parsing Depression From Fatigue in Patients with Cancer Using the Fatigue Symptom Inventory. *Journal of Pain and Symptom Management*, *42*(1), 52–59. https://doi.org/10.1016/j.jpainsymman.2010.10.262

52. Tsatsou, I., Parpa, E., Tsilika, E., Katsaragakis, S., Batistaki, C., Dimitriadou, E., & Mystakidou, K. (2019). A Systematic Review of Sexuality and Depression of Cervical Cancer Patients. *Journal of Sex & Marital Therapy*, *45*(8), 739–754. https://doi.org/10.1080/0092623X.2019.1610125

53. Vistad, I., Fosså, S. D., Kristensen, G. B., & Dahl, A. A. (2007). Chronic fatigue and its correlates in long-term survivors of cervical cancer treated with radiotherapy. *BJOG: An*

International Journal of Obstetrics and Gynaecology, *114*(9), 1150–1158. https://doi.org/10.1111/j.1471-0528.2007.01445.x

54. Vorn, R., Touch, S., & Ryu, E. (2019). Depression and health-related quality of life among Cambodian patients with cancer. *The International Journal of Health Planning and Management, 34*(4), e1747–e1759. https://doi.org/10.1002/hpm.2888
55. Wen, Q., Shao, Z., Zhang, P., Zhu, T., Li, D., & Wang, S. (2017). Mental distress, quality of life and social support in recurrent ovarian cancer patients during active chemotherapy. *European Journal of Obstetrics, Gynecology, and Reproductive Biology*, *216*, 85–91. https://doi.org/10.1016/j.ejogrb.2017.07.004
56. Widyastuty, A., Effendy, E., & Amin, M. M. (2019). Correlation between Visual Analogue Scale Score and Hospital Anxiety Depression Scale-Depression Score in Patients with Cervical Cancer in the Hospital Vina Cancer, Medan. *Open Access Macedonian Journal of Medical Sciences*, *7*(16), 2634–2637. https://doi.org/10.3889/oamjms.2019.473
57. Yang, Y.-L., Liu, L., Wang, X.-X., Wang, Y., & Wang, L. (2014). Prevalence and Associated Positive Psychological Variables of Depression and Anxiety among Chinese Cervical Cancer Patients: A Cross-Sectional Study. *PLOS ONE*, *9*(4), e94804. https://doi.org/10.1371/journal.pone.0094804
58. Ye, S., Yang, J., Cao, D., Lang, J., & Shen, K. (2014). A systematic review of quality of life and sexual function of patients with cervical cancer after treatment. *International Journal of Gynecological Cancer: Official Journal of the International Gynecological Cancer Society*, *24*(7), 1146–1157. https://doi.org/10.1097/IGC.0000000000000207
59. Yoo, H., Shin, D. W., Jeong, A., Kim, S. Y., Yang, H.-K., Kim, J. S., Lee, J. E., Oh, J. H., Park, E.-C., Park, K., & Park, J.-H. (2017). Perceived social support and its impact on depression and health-related quality of life: A comparison between cancer patients and

general population. *Japanese Journal of Clinical Oncology*, *47*(8) , 728– 734. https://doi.org/10.1093/jjco/hyx064

Figure1

Click here to access/download;Figure;figure1.tif

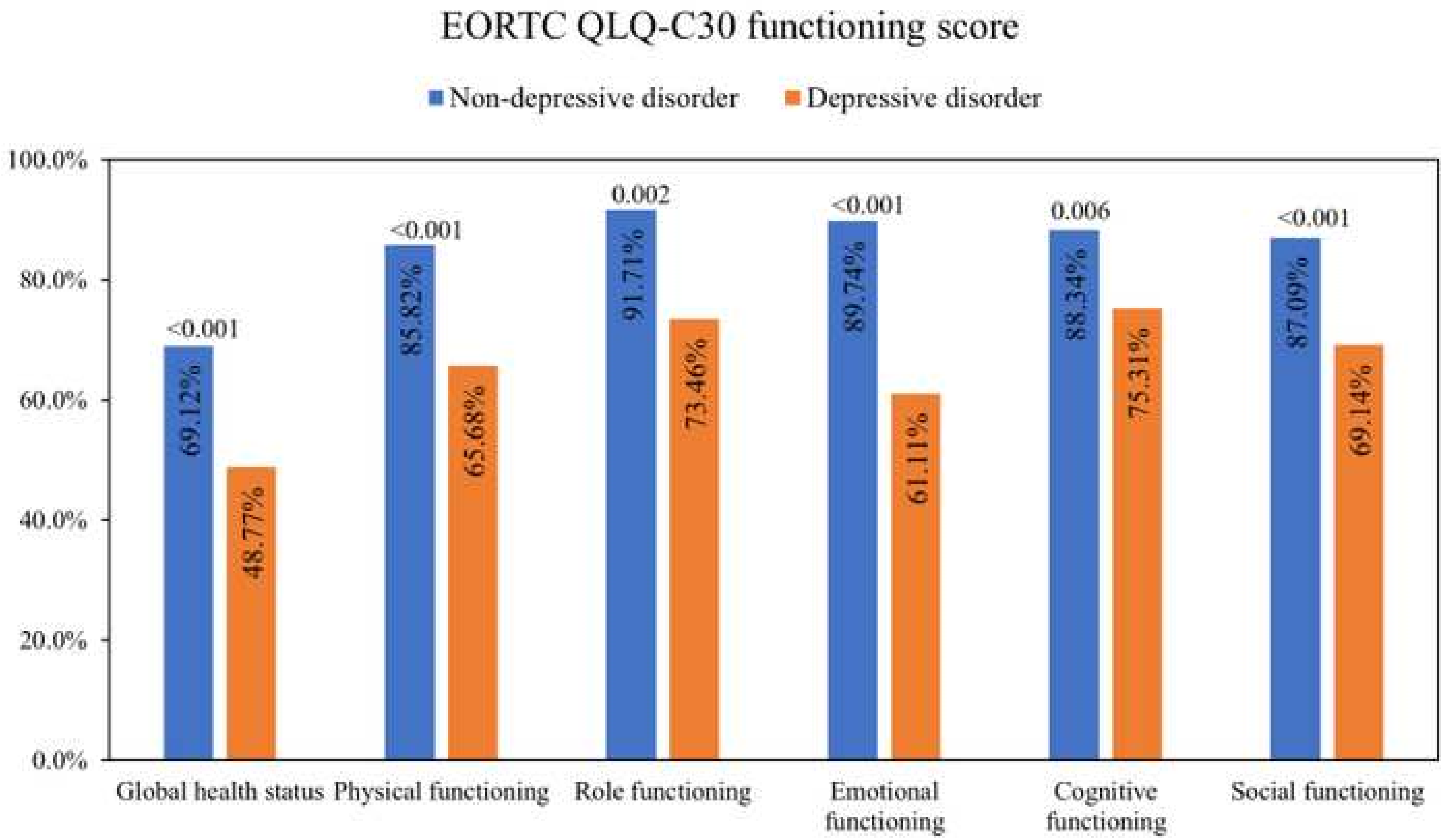

Figure2

Click here to access/download;Figure;figure2.tif

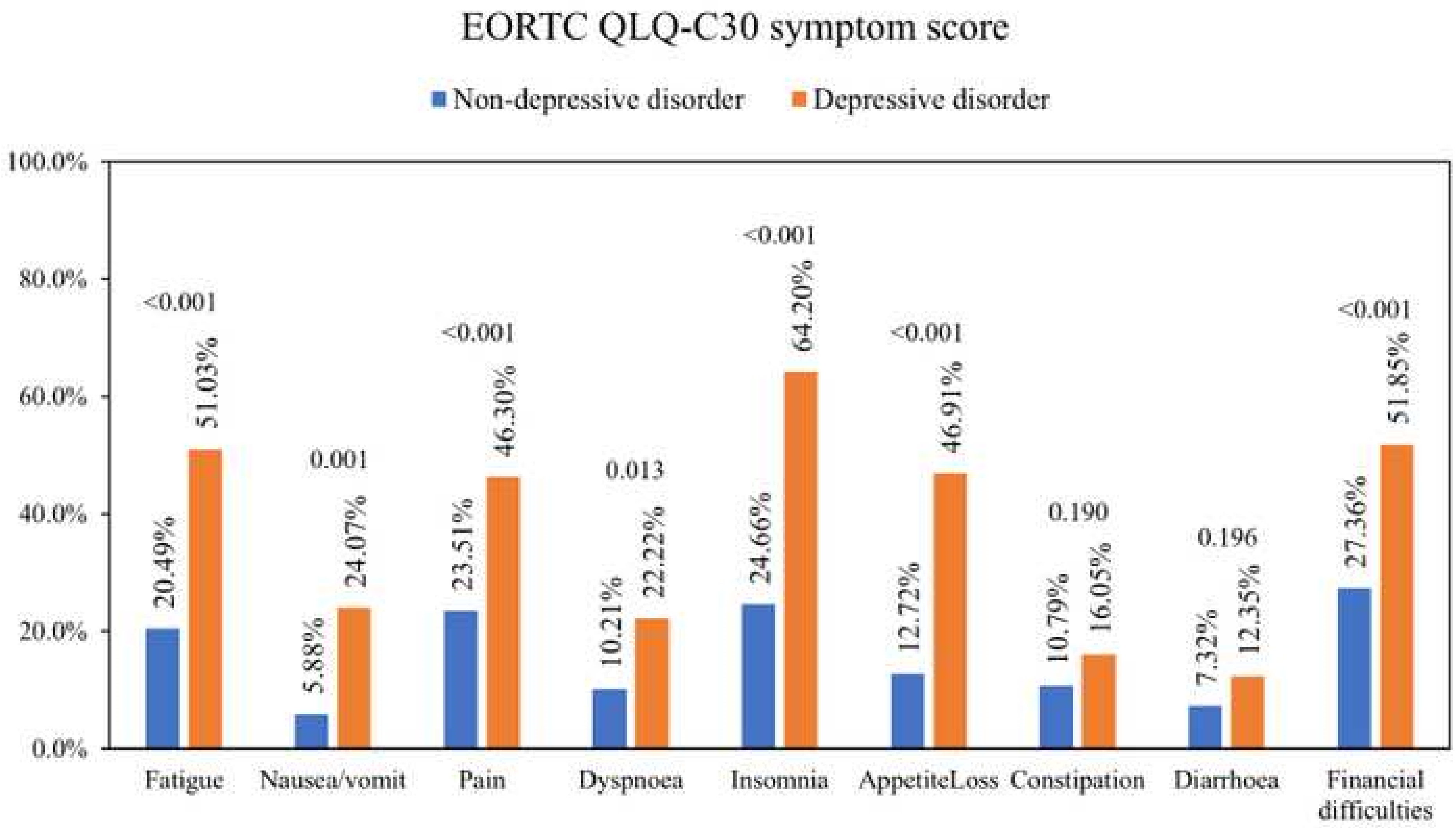

Figure3

Click here to access/download;Figure;figure3.tif

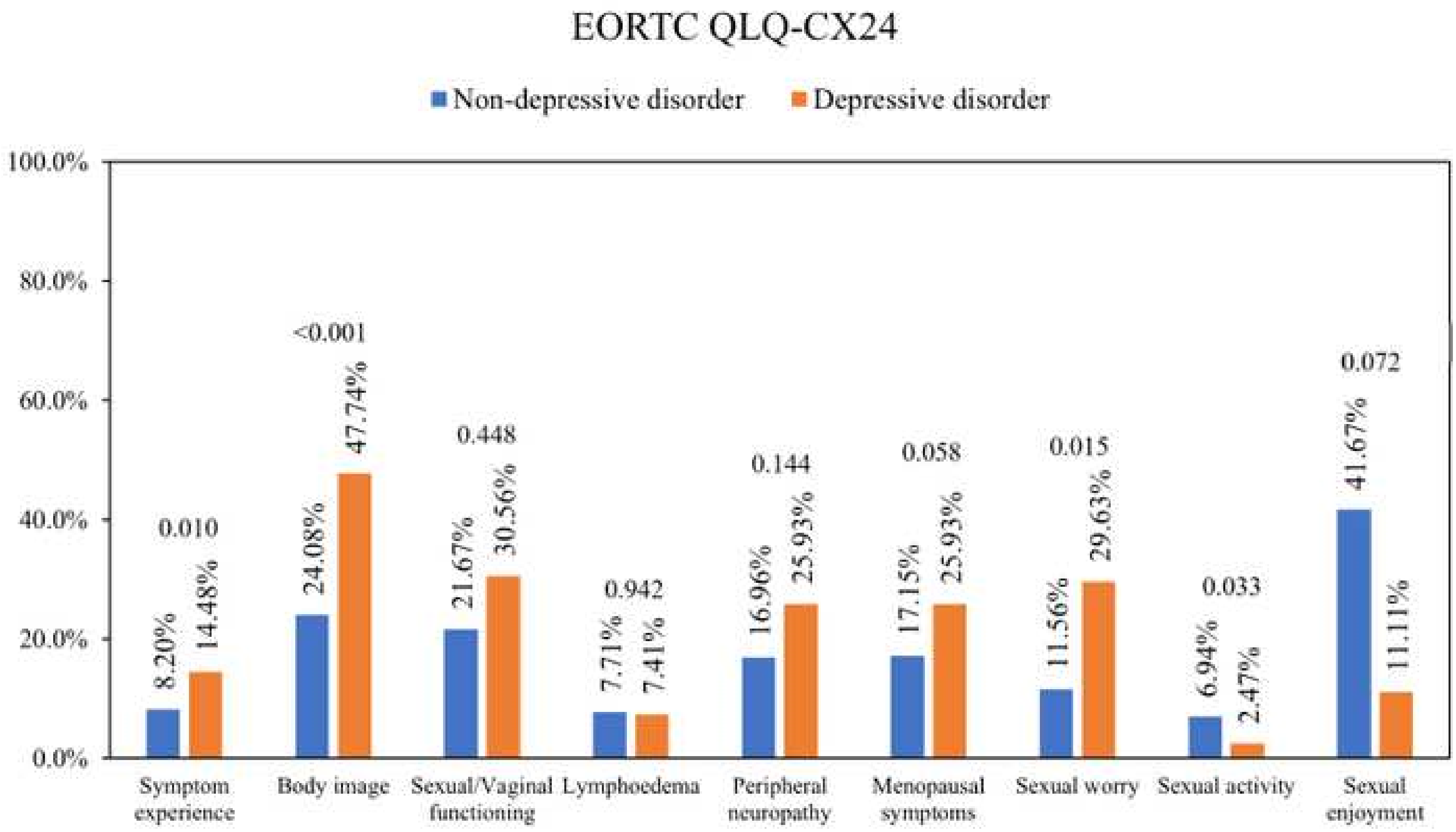

Supplement: Attachment — Submitted filename: PONE-D-20-32916_reviewer (1).pdf [file pone.0252779.s003.pdf]
